# Accuracy of institutional orthopedic trauma databases: a retrospective chart review

**DOI:** 10.1186/s13018-021-02478-3

**Published:** 2021-06-07

**Authors:** Aman Chopra, Abigail C. Cortez, Ashraf El Naga, Anthony Ding, Saam Morshed

**Affiliations:** 1 Georgetown University School of Medicine, 3900 Reservoir Road, NW, Washington, DC 20007 USA; 2 UCLA Department of Orthopaedic Surgery, 10833 LeConte Avenue, 76-119 CHS, Los Angeles, CA 90095-6902 USA; 3 Orthopaedic Trauma Institute, UCSF Department of Orthopaedic Surgery, 2550 23rd St, San Francisco, CA 94110 USA

**Keywords:** Trauma database, Trauma registry, Database accuracy, Resident database

## Abstract

**Introduction:**

Academic trauma institutions rely on fracture databases as research and quality control tools. Frequently, these databases are populated by trainees, but the completeness and accuracy of such databases has not yet been evaluated. The purpose of this study is to determine the capture rate of a resident-populated database in collecting extremity fractures and to determine the accuracy of assigned Orthopaedic Trauma Association (OTA) classifications.

**Materials and methods:**

A retrospective study was performed at a level 1 trauma center of all adult patients who underwent treatment for extremity fractures after an emergency department or inpatient consultation. A 20% random sample was taken from these entries and compared to a resident-populated fracture database designed to capture the same patients. For all matching records containing a resident-assigned OTA classification, relevant imaging was blindly reviewed by a trauma fellowship-trained orthopedic attending surgeon for fracture pattern classification. Resident OTA classifications were compared to this gold standard to determine overall accuracy rate.

**Results:**

Three hundred eighteen (80%) out of 400 entries were captured by the resident-populated database. Two hundred thirty-one of these 318 entries contained an OTA classification. One hundred fifty-three (66%) of these 231 entries demonstrated concordance between resident and attending assigned OTA classifications. On subgroup analysis, 133 (70%) of the 190 lower extremity classifications were accurately identified as compared to just 20 (49%) of the 41 upper extremity classifications (*p* = 0.009). Seventy-nine (65%) of the 121 end segment fractures showed agreement versus 42 (67%) of the 63 diaphyseal injury patterns (*p* = 0.85). Accuracy of classification did not significantly vary by resident year of training (*p* = 0.142).

**Conclusion:**

Trainee generated databases at academic institutions may be subject to incomplete data entry and inaccurate fracture classifications. Quality control measures should be instituted to ensure accuracy in such databases if efforts are invested with the expectation of useful information.

## Introduction

The development and maintenance of trauma registries has been essential in the advancement of high-quality trauma care delivery. These extensive acute care databases capture detailed information on epidemiology, mechanism of injury, and trauma care outcomes [1–4]. Trauma systems have relied on trauma registries for over 30 years and such databases have proven to be extremely valuable for research purposes and quality control [5].

The effectiveness of trauma registries to improve clinical research and patient outcomes depends on the quality of data recorded. If the data captured in the registry is incomplete or inaccurate, comparing trauma care systems will be ineffective and utilizing trauma care outcome data will not be beneficial [6]. In addition to poor data quality, high costs associated with managing a trauma database is also an important consideration. In 2015, the cost associated with managing single hospital trauma registries globally was valued between $10,000 and $1 million (USD) [7]. Trauma registry development and maintenance requires substantial financial costs and well-trained staff to gather and analyze data. The inadequate number of staff members with vast knowledge in trauma epidemiology and outcome research further limits the utility of such a database [8].

Utilizing orthopedic surgery resident physicians as a means of capturing accurate and complete data on trauma patients and outcomes can be a cost-effective way to build a valuable institutional trauma registry for research purposes. The completeness and accuracy of such trainee-populated databases at academic trauma institutions has not yet been evaluated. The purpose of this study is to determine the completeness of a resident-populated trauma database in collecting information on all upper and lower extremity fractures treated at a single institution and to determine the accuracy of resident-assigned Orthopedic Trauma Association (OTA) classifications.

## Materials and methods

This retrospective cohort study was conducted at a single university-affiliated public level 1 trauma center and was approved by the institutional review board. Using appropriate Current Procedural Terminology (CPT) codes, we queried our institution’s main orthopedic billing database for adult patients greater than 18 years of age who sustained an upper extremity or lower extremity fracture and received definitive treatment at our hospital between April 2012 and February 2017. A 20% random sample was obtained from these entries, and medical chart review of inpatient documentation and radiographs was undertaken to confirm eligibility. All nonunions, peri-prosthetic fractures, and dislocations were excluded from analysis. Included entries were compared to a resident-populated orthopedic trauma database designed to capture the same patient population and record all trauma injuries presenting to our institution. Residents were tasked with capturing incoming patients with upper or lower extremity fractures and classifying injuries based on the Orthopaedic Trauma Association (OTA) system for bone, segment, and fracture type. The residents participating in the study were either on their orthopedic trauma rotation or taking trauma call at the academic level 1 trauma center. Participating residents were trained on orthopedic trauma database utilization by the clinical research coordinator who regularly reminded participants to record patient extremity fractures during weekly fracture conferences. To ensure that patients from the billing and resident databases were identical, matching was done based on medical record number, date of admission, and affected bone.

For all matched records containing a resident-assigned OTA classification, relevant imaging was reviewed blindly by a trauma fellowship-trained orthopedic surgeon for correct classification of fracture patterns. Resident OTA classifications were compared to this gold standard to determine overall accuracy rate of the resident database. Subgroup analyses were also performed with chi-squared tests to determine whether accuracy rates for upper extremity versus lower extremity and end segment versus diaphysis classifications were significantly different. Additional subgroup analysis was also performed to determine accuracy of classification by comparing resident year levels of training, which included postgraduate years one to three.

## Results

**Fig. 1:**
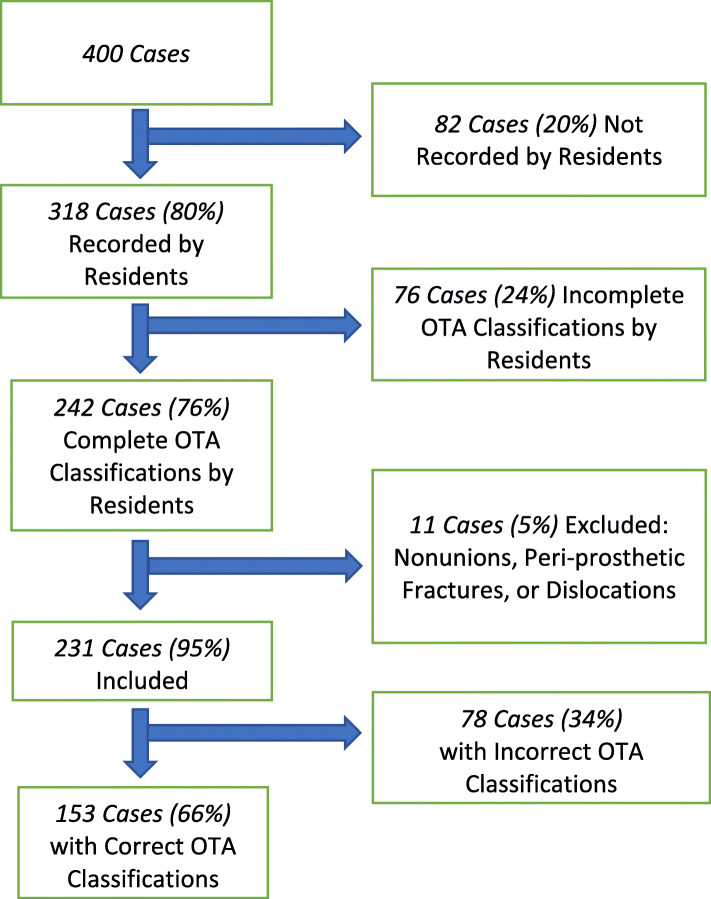
Flow chart of 400 patient records analyzed for completeness and accuracy

Between April 2012 and February 2017, 2002, unique fracture records were identified from the main orthopedic billing database. Representing a 20% sample, 400 entries were randomly selected and compared to the orthopedic database. Three hundred eighteen (80%) out of 400 entries, comprised of emergency department visits and inpatient orthopedic consultations, were captured by residents (Fig. 1). Two hundred forty-two (76%) out of these 318 resident entries contained a completed OTA classification, and imaging for these patients was blindly reviewed for appropriate OTA classification by an experienced surgeon. The remaining 24% of entries had incomplete or absent OTA classifications assigned by residents. Prior to accuracy analysis, 11 entries were excluded because injury patterns were described as nonunions, peri-prosthetic fractures, or dislocations. One hundred fifty-three of the remaining 231 entries demonstrated congruence in OTA classification between resident and attending assignments for an overall accuracy rate of 66% (Fig. 1).

**Fig. 2:**
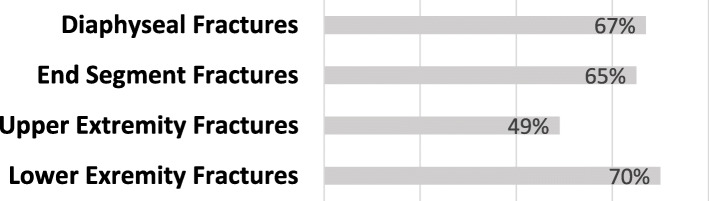
Percentage of accurate OTA classifications for different fracture subgroups

On subgroup analysis, 133/190 (70%) lower extremity classifications were accurately identified compared to just 20/41 (49%) upper extremity classifications (*p* = 0.009). Seventy-nine (65%) of the 121 end segment fractures showed agreement versus 42 (67%) of the 63 diaphyseal injury patterns (*p* = 0.85) (Fig. 2). Accuracy of classification did not significantly vary by resident year of training (*p* = 0.142).

## Discussion

Trauma registries serve as an important research and quality control tool for academic hospitals. Physician residents can aid in data collection for trauma databases, with the aim of reducing registry operating costs and errors in data entry. Both the completeness and accuracy of trainee-populated databases has not yet been investigated. The purpose of this study is twofold: (1) to determine the capture rate of a resident-populated database at collecting information on all upper and lower extremity fractures treated at a single institution and (2) to determine the accuracy of resident-assigned Orthopaedic Trauma Association (OTA) classifications. The residents at our institution were able to capture 80% of a randomly selected cohort of 400 cases treated at the level 1 trauma center, while correctly assigning OTA classification for 66% of the captured cases. Specifically, 70% of lower extremity fractures were correctly classified as opposed to 49% of the upper extremity fractures. Resident year did not show a significant correlation with accuracy of classification.

O’Reilly and colleagues conducted a literature review on 69 publications discussing trauma registries to determine the different methods used in classification, measurement, and improvement of data quality in trauma databases. Most studies addressed at least one of the 3 main data quality dimensions—capture rate, accuracy, and completeness, while our study discussed all factors to some degree [9]. Literature on registries discuss the importance of professional paid staff to abstract and validate patient data [7, 10–12]. Wynn et al. compared data from trauma patients from both trauma and administrative databases based on matching ICD-9 codes at a level 1 trauma center. The medical record and billing personnel group underreported mechanism of injury, diagnoses, diagnostic interventions, surgical procedures, and complications from orthopedic injuries as compared to trained trauma registry staff (1243 correct entries vs. 1101 correct entries) [11]. This suggests that trauma registries populated by staff well-versed in clinical aspects of trauma care can potentially be more complete and detailed than using administrative staff to record data for research and quality control purposes.

O’Reilly et al. published the first survey of trauma registry custodians, working for single hospital and multi-hospital registries across the globe. From the 40 single hospital registries, more than half employed at least one person for each job description: director, manager, data manager, trauma nurse coordinator, and data collector (primarily nurses with no clinical duties). Thirty of these registries had a total operating cost between $10,000 and $1 million per year (USD) to manage entry of more than 100 data elements [7]. Our study shows how a trainee-populated registry can be more accurate and complete than a database populated by administrative staff specialized in medical records and billing. Further, utilizing a resident database is likely to be less costly to maintain for a single institution.

Most of the research on registries specific to orthopedic trauma has been investigated in the context of the US military. Specifically, The Military Orthopedic Trauma Registry (MOTR), which began live data abstraction in 2013, compiles detailed information on war injuries with the aim of improving clinical practice guidelines for extremity injury combat casualty care [13–15]. Rivera et al. conducted a quality assurance survey of MOTR entrants for lower extremity injuries to determine if this data could provide robust orthopedic trauma information for late amputation causes. The study found that 80% of 45 entries listed the direct contributing factor (infection, nonunion, etc.) to late amputation, suggesting that this fracture database is detailed enough to answer clinically relevant questions for orthopedic trauma surgeons [16]. Similar to how this study showed 80% data completeness for amputation causes, our study showed 231/318 (73%) complete OTA classifications for both upper and lower extremity injuries, suggesting that an institutional trauma database run by trainees can be just as complete as those run by trauma registrars from MOTR.

This study does have limitations. First, the study was conducted at only one institution and therefore it is uncertain whether the results are generalizable to other trauma centers. Second, the data was abstracted and interpreted by physician residents. The trainee population mainly included first, second, and third year residents, and thus may have reduced the accuracy and completeness for data entry due to resident inexperience. Fourth and fifth year residents were mainly unavailable to participate in the study due to time constraints. Lastly, a sample size of only 400 out of 2002 original entries was analyzed and therefore could have impacted the results of the capture rate and OTA classification accuracy. In a future study, more of the original entries should be analyzed to confirm relative accuracy and completeness. Moving forward, a large multi-center study in which the resident population is equally distributed across all five resident levels may be required to validate study findings. The current study highlights the necessity to implement a regimented process of reviewing the registry data to ensure quality. Institutions that maintain large internal databases should allocate resources to have a clinical research coordinator confirm data completeness and have an experienced orthopedic surgeon consistently evaluate the accuracy of the abstracted data.

## Conclusion

Trainee-generated databases at academic institutions may be subject to incomplete data entry and inaccurate fracture classifications. Quality control measures should be instituted to ensure accurate data in such databases if time and effort are invested with the expectation of a useful databank and research tool.

## Data Availability

The datasets used and/or analyzed during the current study are available from the corresponding author on reasonable request.

## Abbreviations

OTA: Orthopedic trauma classifications
USD: United States dollars
CPT: Current procedural terminology
ICD-9: International Classifications of Disease, Ninth Revision
MOTR: Military Orthopedic Trauma Registry
